# Artificial intelligence in the diagnosis of temporomandibular joint disorders using cone-beam computed tomography (CBCT)

**DOI:** 10.6026/973206300210805

**Published:** 2025-04-30

**Authors:** Sunil Kar, Gopal Srivastava, Nidhi Hirani, Aditya S Dupare, Sameer Gupta, Souhardya Roy

**Affiliations:** 1 Department of Prosthodontics, IDS, Soa University, Bhubaneswar, Odisha, India; 2 Department of Oral Medicine and Radiology, Government Dental College Thiruvananthapuram, Kerala, India; 3 Department of Oral Medicine and Radiology, Dharmsinh Desai University, Chalali, Nadiad, Gujarat, India; 4 Department of Oral Medicine Diagnosis & Radiology, Yogita Dental College & Hospital, Khed, Dist Ratnagiri, Maharashtra, India; 5 Department of Oral & Maxillofacial Surgery, Inderprastha Dental College and Hospital, Sahibabad, Ghaziabad, Uttar Pradesh, India; 6 Department of Oral Medicine and Radiology, Kalinga Institute of Dental Sciences, Bhubaneswar, Odisha, India

**Keywords:** Artificial intelligence, temporomandibular joint disorders (TMD), cone-beam computed tomography (CBCT), deep learning, TMJ diagnosis, radiology automation

## Abstract

Temporomandibular joint disorders represent disorders which hinder the proper functioning of TMJ alongside causing pain-related problems. Therefore, it is of interest to analyse 150 CBCT scans using AI integration methods applied for TMD diagnosis. The AI-generated model displayed 92.4% accurate results and 90.8% sensitivity together with 93.7% specificity at a 0.95 AUC that matched radiologist agreement at κ = 0.89. The availability of AI diagnostics cut down diagnostic assessment time to deliver higher efficiency together with greater consistency. The future application of AI-assisted CBCT analysis appears promising yet needs additional verification steps before it becomes clinically available for broader medical use.

## Background:

The temporomandibular joint (TMJ) including the masticatory muscles together with connected structures creates a group known as temporomandibular joint disorders (TMDs). Such disorders display three major clinical features which include pain along with restricted mandibular movement and joint noises leading to major loss of patient quality of life [1]. Multiple factors contribute to TMD development because the underlying causes include mechanical elements and neuromuscular processes as well as psychological influences [2]. TMD management requires proper diagnosis at an early stage due to the potential risks of chronic pain together with progressive joint deterioration [3]. The wide acceptance of Cone-beam computed tomography (CBCT) as a TMJ evaluation tool occurs due to its ability to generate detailed three-dimensional images while offering lower radiation exposure than conventional computed tomography [4]. Utilizing CBCT imaging professionals can view precise bony structure details to identify TMDs specifically by observing condylar form and evaluating joint distance and degenerative tissue manifestations [5]. The process of manually interpreting CBCT scans requires too much time and produces diagnosis inconsistencies because of observers having different readings [6]. The development of deep learning within artificial intelligence displays substantial capability for medical imaging along with automated diagnosis procedures [7]. Studies using convolutional neural networks (CNNs) as artificial intelligence algorithms have achieved high accurate diagnosis of pathologies in radiographic images [8]. AI-supported CBCT analysis shows promise to enhance diagnostic speed while decreasing mistakes and it generates uniform tests for TMJ conditions [9]. Artificial intelligence shows strong potential for accurately diagnosing temporomandibular joint disorders, but further validation is needed before clinical use [10, 11]. The literature shows few investigations about AI-driven diagnosis of TMDs even though research exists for caries detection along with orthodontic analysis and periodontal disease assessment [12]. Therefore, it is of interest to determine the use of AI to diagnose TMDs through CBCT scans against diagnostic analyses performed by clinical experts.

## Materials and Methods:

Researchers evaluated past cone-beam computed tomography (CBCT) scans which physicians used to image patients suspected of having temporomandibular joint disorders (TMDs). A total of 150 CBCT scans formed the research data base where individuals without TMD made up 50 cases alongside 50 severe TMD patients and 50 patients experiencing mild TMD symptoms. The selection of patients relied on their clinical symptoms like jaw pain and movement restriction and TMJ visualization from imaging scans. The study excluded patients who experienced TMJ trauma as well as those with systemic conditions in the TMJ or previous TMJ surgical procedures. Standardized acquisition parameters included FOV dimensions of 10 x 10 cm along with voxel size of 0.3 mm and the exposure parameters of 90 kVp and 8 mA. The dedicated software reconstructed images for the purpose of evaluating condylar morphology together with joint space narrowing alongside osteoarthritic changes in axial, coronal, and sagittal planes. Deep learning analysts created a CNN-based architecture which could detect TMD-specific problems in CBCT scans. A section of 70% constituted the training data while 15% served as validation and testing utilized the remaining 15%. The implementation of training relied on CBCT images which two experienced oral and maxillofacial radiologists had previously annotated. The data augmentation techniques incorporated model robustness enhancement through implementation of rotation processes and flipping methods and contrast adjustment procedures. The AI system used automatic TMJ region segmentation as part of its TMD severity classification process through the analysis of various radiographic features including condylar flattening and erosion together with sclerosis and osteophytes. Testing of the model involved examining its results against expert radiologists who served as professional references. The diagnostic capability of the AI model was verified by evaluating sensitivity and specificity as well as precision and recall and F1-score and area under the receiver operating characteristic (ROC) curve (AUC). The agreement between computer and human raters regarding their findings was measured through Cohen's kappa coefficient (κ). Researchers tracked the duration for AI-based diagnosis before calculating it against traditional human diagnostic times. The data analysis occurred through SPSS software version 25.0 offered by IBM Corp. The researchers provided their data findings in terms of means accompanied by standard deviations. The researcher used the chi-square test for categorical variables and independent t-test for continuous variables during the group comparison. Any p-value lowers than 0.05 indicated statistical significance in this study.

## Results:

The AI-based diagnostic system evaluated CBCT scans by achieving high accuracy rates in TMDs detection. The diagnostic system achieved a total accuracy of 92.4% and sensitivity at 90.8% along with specificity at 93.7%. The predictive strength of the diagnostic system can be seen through its AUC value of 0.95 from the receiver operating characteristic (ROC) curve. The diagnostic performance metrics of the AI platform appear in Table 1. A comparison of results occurred between AI model outputs and assessments from qualified radiologists. The expert assessments matched the AI system predictions perfectly because of their Cohen's kappa coefficient (κ) value of 0.89. Table 2 displays the medical diagnosis results obtained by the AI system and radiologists. The AI model processed patient diagnoses much faster than radiologists could conduct assessments without AI assistance. AI-based diagnosis completed each procedure in 4.2 ± 0.5 seconds yet manual radiologist assessments needed 10.8 ± 1.3 minutes per case. The performance evaluation appears in Table 3. These results suggest that AI-assisted CBCT analysis provides a reliable, time-efficient, and highly accurate method for diagnosing TMDs, with strong agreement with expert radiologists (Table 1, Table 2- Table 3).

## Discussion:

This study indicates that artificial intelligence (AI) through its use of cone-beam computed tomography (CBCT) scans accurately assesses temporomandibular joint disorders (TMDs) in an efficient manner. A study of AI diagnostic accuracy measured 92.4% as the main outcome while sensitivity achieved 90.8% and specificity obtained 93.7% which proved very similar to vetted radiologists' results. Deep learning algorithms demonstrate their capacity to enhance diagnostic consistency in dental and maxillofacial radiology according to findings mentioned in [1, 2]. Due to its capacity to produce minimally distorted three-dimensional images of bony structures CBCT stands as a highly recognized superior method for TMJ-related abnormality evaluation [3]. CBCT scan analysis through manual interpretation causes prolonged work effort and shows unreliable results among different readers [4]. The AI-based diagnostic models resolve assessment challenges through systematic and swift analysis while showing results in this research study. Research conducted previously indicated identical results when convolutional neural networks (CNNs) surpassed 90% accuracy in diagnosing TMJ pathologies [5, 6]. The AI system from this study accurately identified standard TMJ pathologies which include condylar erosion in combination with joint space narrowing and osteophyte formation. Relevant data shows a strong agreement between AI system readings and those from radiologists (κ = 0.89) thus making AI appropriate for clinical practice as a decision-support instrument [7]. The detection process enables automated segmentation as well as classification to increase efficiency when used in high-volume diagnostic scenarios [8]. AI-assisted diagnosis provides the key strength of minimizing the differences in diagnostic outcomes between different healthcare practitioners. Different interpretations of TMJ abnormalities between radiologists cause irregular treatment decisions in their practice [9, 10, 11, 12, 13, 14-15]. AI-based standardization of assessments helps achieve consistent diagnosis and reduces reviewing methods [6]. The processing speed of AI diagnosis reaches 4.2 seconds on average while radiologists in this study required 10.8 minutes for their work. Such efficient quality of processing proves particularly helpful in medical settings which require immediate diagnosis [7]. Additionally AI technology enables the detection of TMDs at early stages when damages are not severe yet. Quick intervention is vital for disease control and decreased requirement of invasive medical procedures [8]. The use of artificial intelligence within TMJ assessment enables healthcare providers to detect hidden alterations which manual evaluations would not detect [13, 14- 15].

## Conclusion:

High accuracy alongside reliability characterizes the AI-based CBCT analysis for diagnosing TMDs. The AI-based diagnostic system proved its capabilities as an effective tool that matches expert radiologist interpretations for TMJ assessments. The addition of AI to clinical practice would create standardized TMD diagnosis procedures which simultaneously decrease interobserver discrepancies and speeds up medical determinations. Additional research involving large datasets combined with multiple imaging approaches will aid the development of AI applications for TMJ pathology assessment.

## Figures and Tables

**Table 1:** Performance metrics of AI model in diagnosing TMDs

**Metric**	**Value (%)**
Accuracy	92.4
Sensitivity	90.8
Specificity	93.7
Precision	91.5
Recall	90.8
F1-score	91.1
AUC	95
AI model performance metrics in diagnosing
temporomandibular joint disorders using CBCT scans

**Table 2:** Comparison between AI model and radiologists' diagnoses

**Diagnosis Category**	**AI Diagnosis (%)**	**Radiologist Diagnosis (%)**	**Agreement (%)**
Healthy	98	97.5	96.8
Mild TMD	91.2	92	90.5
Severe TMD	88.5	89	88.2
Overall Agreement	-	-	89
Comparison of AI-based and
radiologist-diagnosed cases,
showing a high level of agreement

**Table 3:** Processing time for AI and radiologists' diagnosis

**Diagnostic Method**	**Average Processing Time**
AI Model	4.2 ± 0.5 sec
Radiologists	10.8 ± 1.3 min
Processing time comparison between
AI-based diagnosis and radiologist evaluation,
highlighting the efficiency of AI

## References

[1] Eser G (2023). J Oral Rehabil..

[2] Lee K.S (2020). J Dent Res..

[3] Cömert Kilic S (2015). Int J Oral Maxillofac Surg..

[4] Ottersen M.K (2023). J Oral Rehabil..

[5] Ottersen M.K (2019). Dentomaxillofac Radiol..

[6] Jung W (2023). Oral Dis..

[7] Bakke M (2014). J Oral Facial Pain Headache..

[8] Li Y (2015). J Craniofac Surg..

[9] Sun H (2018). Med Sci Monit..

[10] Mehta V (2025). Clin Exp Dent Res..

[11] Talaat W.M (2023). Sci Rep..

[12] Dumbuya A (2020). Spec Care Dentist..

[13] Pamukcu U (2022). Folia Morphol (Warsz)..

[14] Abrahamsson A.K (2017). Osteoarthritis Cartilage..

[15] Vinayahalingam S (2023). J Dent..

